# Biochemical characteristics and molecular mechanism of an exo-type alginate lyase VxAly7D and its use for the preparation of unsaturated monosaccharides

**DOI:** 10.1186/s13068-020-01738-4

**Published:** 2020-06-01

**Authors:** Luyao Tang, Ying Wang, Shan Gao, Hao Wu, Danni Wang, Wengong Yu, Feng Han

**Affiliations:** 1 Key Laboratory of Marine Drugs, Ministry of Education; Shandong Provincial Key Laboratory of Glycoscience and Glycoengineering, School of Medicine and Pharmacy, Ocean University of China, 5 Yushan Road, Qingdao, 266003 China; 2 Laboratory for Marine Drugs and Bioproducts of Qingdao National Laboratory for Marine Science and Technology, Qingdao, 266237 China; 3 Biology Institute, Qilu University of Technology (Shandong Academy of Sciences), Jinan, 250103 Shandong China

**Keywords:** Alginate, Alginate lyase, Exo-type, Polysaccharide lyase, Monosaccharide, 4-Deoxy-l-*erythro*-5-hexoseulose uronate (DEH), *Vibrio*

## Abstract

**Background:**

As the most abundant polysaccharide in brown algae, alginate has become a promising economical material for bioethanol production. Recently, exo-type alginate lyases have received extensive attention because the unsaturated monosaccharides produced by their degradation of alginate can be easily converted into 4-deoxy-l-*erythro*-5-hexoseulose uronate (DEH), a promising material for bioethanol production and biorefinery systems.

**Results:**

In this study, we cloned and characterized an exo-type polysaccharide lyase family 7 (PL7) alginate lyase VxAly7D from the marine bacterium *Vibrio xiamenensis* QY104. Recombinant VxAly7D was most active at 30 °C and exhibited 21%, 46% and 90% of its highest activity at 0, 10 and 20 °C, respectively. Compared with other exo-type alginate lyases, recombinant VxAly7D was shown to be a bifunctional alginate lyase with higher specific activity towards sodium alginate, polyG and polyM (462.4 ± 0.64, 357.37 ± 0.53 and 441.94 ± 2.46 U/mg, respectively). A total of 13 μg recombinant VxAly7D could convert 3 mg sodium alginate to unsaturated monosaccharides in 1 min with a yield of 37.6%, and the yield reached 95% in 1 h. In addition, the three-dimensional structure of VxAly7D was modelled using the crystal structure of AlyA5 from *Zobellia galactanivorans* Dsij^T^ as the template. The action mode and the end products of the W295A mutant revealed that Trp^295^ is a key amino acid residue responsible for the exolytic action mode of VxAly7D.

**Conclusion:**

Overall, our results show that VxAly7D is a PL7 exo-type alginate lyase with high activity and a high conversion rate at low/moderate temperatures, which provides a useful enzymatic tool for the development of biofuel production from brown algae and enriches the understanding of the structure and functional relationships of polysaccharide lyases.

## Background

With the demand for energy growing constantly and fossil fuel resources being depleted, bioethanol has received more attention as renewable, clean energy [1–3]. Brown algae have become an important source of bioethanol production because of showing excellent characteristics including a wide distribution, high productivity, and absence of lignin, without requiring arable land, freshwater, or fertilizer [4–6].

Alginate is the major polysaccharide of the cell wall in brown algae, which may account for 40% of the dry weight of brown algae [7, 8]. Alginate is a linear polysaccharide consisting of β-d-mannuronate (M) and its C5 epimer α-l-guluronate (G) connected by 1,4 O-linked glycosidic bonds [9, 10], arranged as poly β-d-mannuronate (polyM), poly α-l-guluronate (polyG) or their heteropolymer (polyMG) [11]. Alginate and its oligosaccharides exhibit high safety and are widely used in food, medicine and industrial production [12].

Alginate lyase degrades alginate via the β-elimination reaction, producing unsaturated C=C double bonds between C4 and C5 at the nonreducing end [13]. Thousands of alginate lyases have been discovered, and more than a hundred of these enzymes have been characterized. However, only a few of these enzymes show high activity at low/moderate temperatures [14–20]. Alginate lyases with high activity and stability at low/moderate temperatures are suitable for many industrial productions purposes because they can be selectively inactivated by slightly increasing the temperature, which can save energy and reduce biological pollution [17, 21–23]. According to substrate specificities, alginate lyases are classified into polyM-specific lyases (EC 4.2.2.3), polyG-specific lyases (EC 4.2.2.11) and bifunctional lyases (EC 4.2.2.-). Based on amino acid sequence analysis, alginate lyases are classified into 12 polysaccharide lyase (PL) families (PL5, 6, 7, 14, 15, 17, 18, 31, 32, 34, 36, and 39) in the Carbohydrate-Active enZYmes (CAZy) database (http://www.cazy.org/). Based on differences in the action mode, alginate lyases can be further divided into endo- and exo-types [24]. Most of the known alginate lyases are of the endo-type and degrade alginate into unsaturated oligosaccharides with different degrees of polymerization (DP) [25–27]. Exo-type alginate lyases are classified as PL6, 7, 14, 15, or 17 family, and degrade alginate polymers and oligomers into unsaturated monosaccharides. The unsaturated monosaccharides are nonenzymatically transformed into 4-deoxy-l-*erythro*-5-hexoseulose uronate (DEH), an essential intermediate in the physiological metabolism of alginate and a potential carbon source for the production of bioethanol and biorefinery [28]. DEH can be reduced to 2-keto-3-deoxygluconate (KDG) by the reductase DehR and feeds into the Entner–Doudoroff (ED) pathway to produce ethanol [4, 6]. Therefore, the degradation of alginate to unsaturated monosaccharides is a crucial step for the production of bioethanol.

However, the specific activity of most currently reported exo-type alginate lyases were approximately 20–70 U/mg [29–34], resulting in a low alginate degradation rate and unsaturated monosaccharide yield. Some of these enzymes even show apparent substrate preferences [32, 35–37], which limits the choice of possible substrates and affects the process of industrialization. To efficiently degrade alginate to produce unsaturated monosaccharides, it has recently been proposed to exploit the synergistic effects of endolytic and exolytic alginate lyases [4, 6, 38, 39]. Nevertheless, the low activity of exo-type alginate lyases is still the rate-limiting step for obtaining unsaturated monosaccharides. Therefore, obtaining exo-type alginate lyases capable of the highly efficient production of unsaturated monosaccharides would be a critical step toward simplifying the construction of engineered strains and promoting the process of industrialization.

In this study, an exo-type PL7 alginate lyase, VxAly7D, from the marine bacterium *Vibrio xiamenensis* QY104 was cloned and characterized. Recombinant VxAly7D efficiently degraded sodium alginate to produce unsaturated monosaccharides at low/moderate temperatures, providing a potentially excellent enzymatic tool for obtaining DEH. In addition, we verified the mechanism of its exo-type action mode, promoting the understanding of the relationship between the structure and function of alginate lyases and enriching the diversity of marine polysaccharide lyases.

## Results

### Cloning and sequence analysis of VxAly7D

**Fig. 1:**
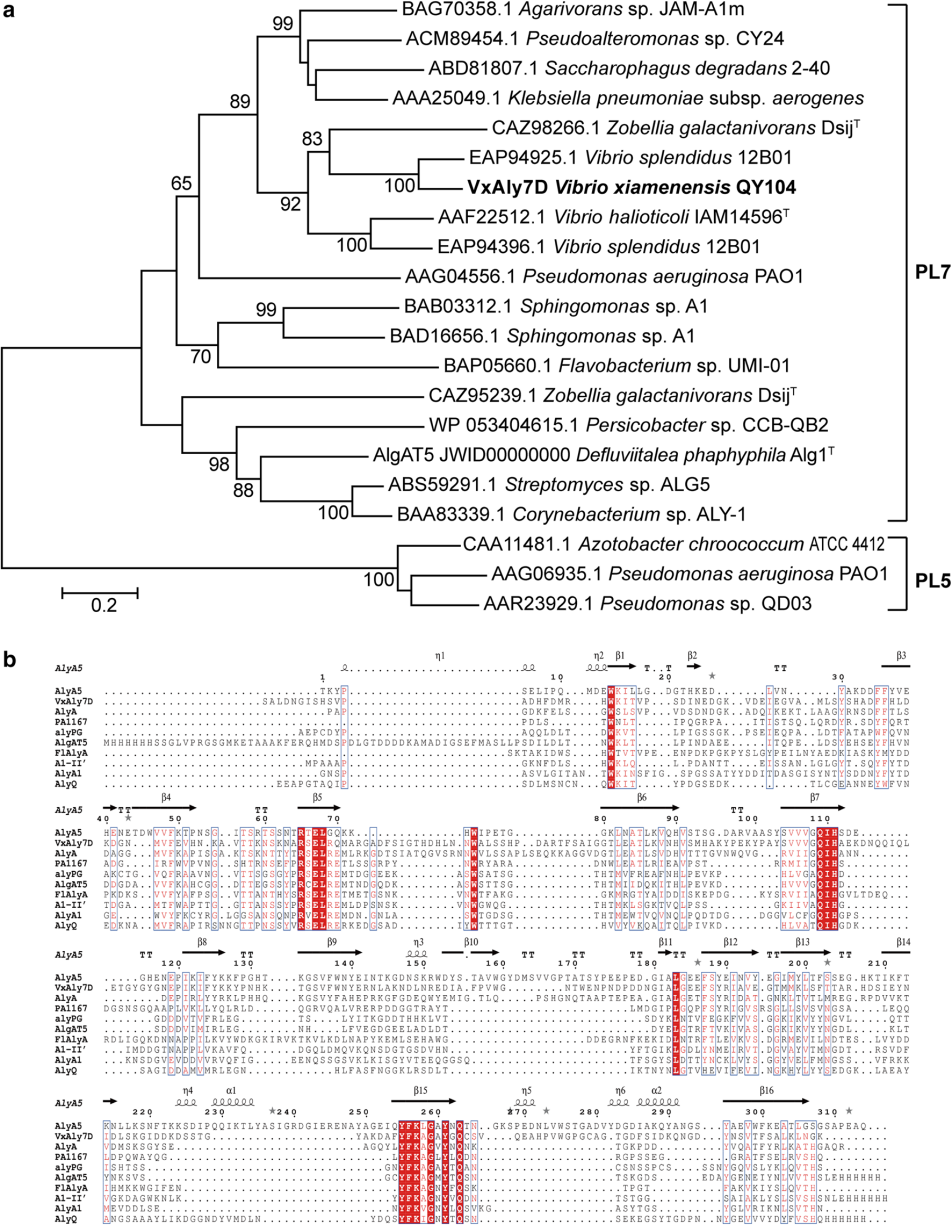
Phylogenetic analysis and multiple amino acid sequence alignment of VxAly7D. **a** Phylogenetic analysis of VxAly7D. The phylogenetic tree was constructed with MEGA 7.0 using the neighbour-joining method. Bootstrap values were expressed based on 1000 replications, the numbers represent bootstrap values (confidence limits) representing the substitution frequencies per amino acid residue, and only values of 70% or above are shown at the nodes. The sequences from this study are shown in bold. **b** Multiple amino acid sequence alignment of VxAly7D with crystallized PL7 enzymes. The secondary structure elements shown above are referenced according to AlyA5. AlyA5, from *Zobellia galactanivorans* Dsij^T^ (CAZ98266.1); AlyA, from *Klebsiella pneumoniae* (AAA25049); PA1167, from *Pseudomonas aeruginosa* PAO1 (AAG04556); alyPG, from *Corynebacterium* sp. ALY-1 (BAA83339); AlgAT5 from *Defluviitalea phaphyphila* Alg1^T^ (JWID00000000); FlAlyA from *Flavobacterium* sp. UMI-01 (BAP05660); A1-II’, from *Sphingomonas* sp. A1 (BAD16656); AlyA1, from *Z. galactanivorans* DsiJ^T^ (CAZ95239); AlyQ from *Persicobacter* sp. CCB-QB2 (WP_053404615)

The genomic sequence analysis of *V. xiamenensis* QY104, an efficient alginate-degrading marine bacterium, revealed six alginate lyase-encoding genes. Four of them belong to the PL7 family, and two belong to the PL17 family. Among these genes, the *vxaly7D* gene is 1041 bp in length and encodes a protein of 346 amino acid residues, including a signal peptide of 19 amino acid residues at the N-terminus. Mature VxAly7D has a calculated molecular mass of 36.46 kDa and a theoretical *pI* of 5.55. According to sequence analysis using BLASTp, VxAly7D showed the highest identity (72.54%) to the characterized PL7 alginate lyase AlyD from *V. splendidus* 12B01. VxAly7D formed a deeply branched cluster with PL7 alginate lyases in the phylogenetic tree (Fig. 1a). Moreover, the alignment of the amino acid sequence of mature VxAly7D with PL7 alginate lyases that had available resolved crystal structures showed that VxAly7D exhibits three highly conserved regions of the PL7 family [40, 41]: R(T/S/C/V)EL(G/R)(E/Q), YFKAGXYXQ, and Q(I/V)H (Fig. 1b). Based on the above analysis, VxAly7D was classified into the PL7 family.

### Heterologous expression and biochemical characterization

**Fig. 2:**
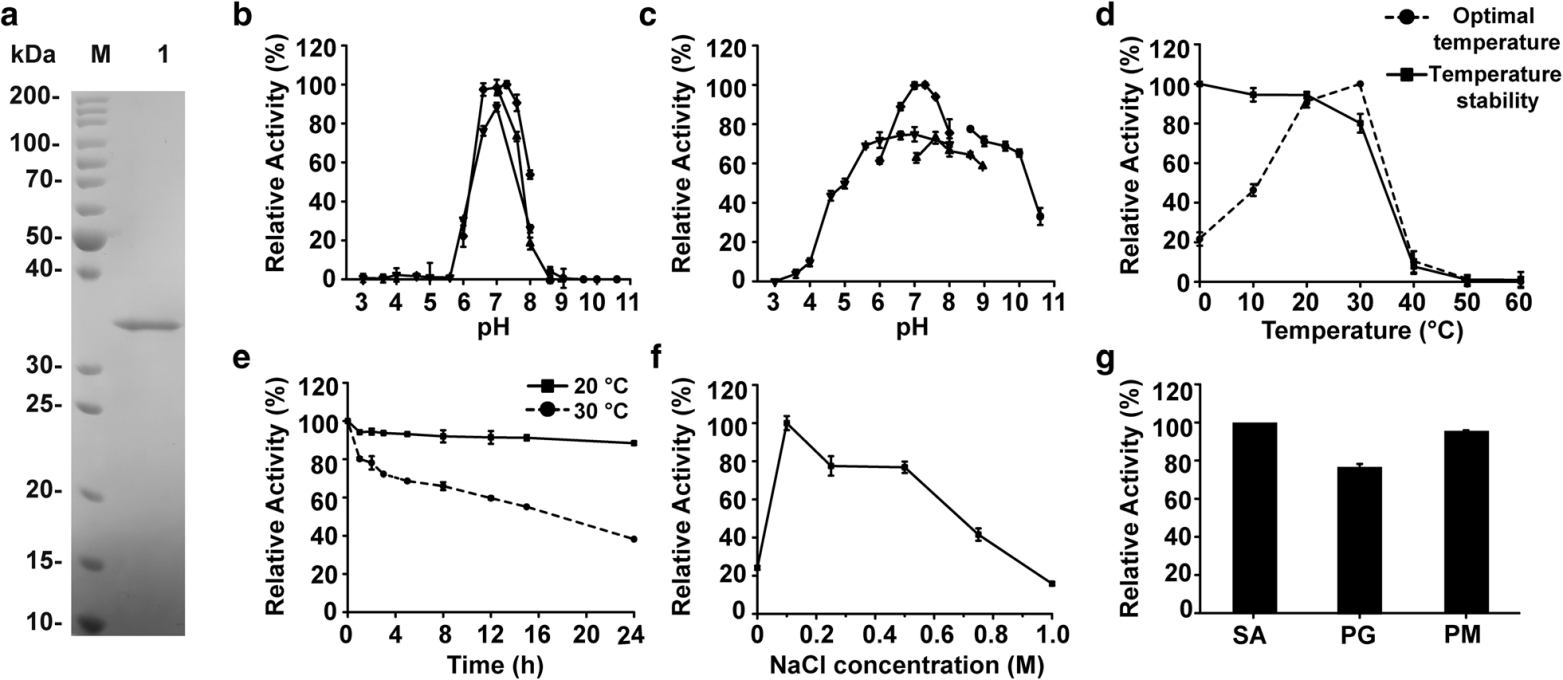
Purity analysis and biochemical characterization of recombinant VxAly7D. **a** Purified recombinant VxAly7D was resolved by 12.5% (w/v) SDS-PAGE. Lane M, Pageruler unstained protein ladder (Thermo Scientific, USA); Lane 1, Purified recombinant VxAly7D. **b** The optimum pH was determined by measuring the activity at 30 °C in 50 mM buffer (Na_2_HPO_4_–citric acid, Na_2_HPO_4_–NaH_2_PO_4_, Tris–HCl, and Gly‐NaOH) at different pH values. The maximum specific activity was 462.4 ± 0.64 U/mg. **c** pH stability. The residual activity was measured at 30 °C in PB (pH 7.3) after incubation at 4 °C for 12 h. The initial specific activity was 438.62 ± 1.22 U/mg. **d** Optimal temperature and temperature stability were determined by measuring its activity at 0–60 °C. The maximum specific activity when measuring the optimum temperature was 462.4 ± 0.64 U/mg. The initial specific activity when measuring the temperature stability was 441.15 ± 2.04 U/mg. **e** The temperature stability of recombinant VxAly7D was measured at 20 °C and 30 °C for 0–24 h. The initial specific activity was 441.15 ± 2.04 U/mg. **f** Effect of the NaCl concentration on enzymatic activity of recombinant VxAly7D. The maximum specific activity was 462.4 ± 0.64 U/mg. g, Substrate specificity towards sodium alginate (SA), polyM (PM) and polyG (PG). The maximum specific activity was 462.4 ± 0.64 U/mg

Recombinant VxAly7D without the predicted signal peptide was heterologously expressed in *Escherichia coli*. The purified recombinant enzyme showed a single band in SDS-PAGE gels with a molecular mass of approximately 36 kDa (Fig. 2a). The activity of recombinant VxAly7D was measured at different pH values (3.0–10.6). Its optimum pH was 7.3 in Na_2_HPO_4_–NaH_2_PO_4_ buffer (PB) (Fig. 2b). The pH stability test for recombinant VxAly7D showed that the enzyme was stable at pH 4.6–10.0, since more than 50% of its activity was retained (Fig. 2c), indicating that recombinant VxAly7D showed broad pH stability. The activity of recombinant VxAly7D was measured at different temperatures (0–60 °C), and the maximum activity was observed at 30 °C (Fig. 2d). Recombinant VxAly7D exhibited 21%, 46%, and 90% of its highest activity at 0, 10 and 20 °C, respectively. Recombinant VxAly7D lost most (92%) of its activity after incubation at 40 °C for 1 h; however, it was stable at 0–30 °C, retaining approximately 80% of its enzyme activity after incubation for 1 h (Fig. 2d). Recombinant VxAly7D still retained 88% of its enzyme activity after incubation for 24 h at 20 °C and 55% of its enzyme activity after incubation for 15 h at 30 °C (Fig. 2e). And the half-life of recombinant VxAly7D was approximately 17 h at 30 °C (Fig. 2e). These results indicated that recombinant VxAly7D presented high activity and stability at low/moderate temperatures.

Recombinant VxAly7D did not require NaCl for enzymatic activity; however, the addition of NaCl significantly enhanced its activity. Recombinant VxAly7D was most active in the presence of 100 mM NaCl (Fig. 2f). The effects of other metal ions, ethylenediaminetetraacetic acid (EDTA), ethylenebis (oxyethylenenitrilo) tetraacetic acid (EGTA), dithiothreitol (DTT) and sodium dodecyl sulfate (SDS) on recombinant VxAly7D activity were also tested. Li^+^, NH_4_^+^, Mg^2+^, Ba^2+^, Ca^2+^, Mn^2+^, and Fe^2+^ could promote its activity, while the other metal ions (Zn^2+^, Ni^2+^, Cu^2+^, Fe^3+^), as well as EDTA, EGTA, DTT and SDS inhibited its activity (Table 1).

**Table 1:** Effect of metal ions, chelators, and detergents on recombinant VxAly7D activity

Reagents added	Concentration	Relative activity (%)
None	0 mM	100.00 ± 2.03
LiCl	1 mM	147.17 ± 2.83
NH_4_Cl	1 mM	156.35 ± 5.23
MgCl_2_	1 mM	108.27 ± 2.50
CaCl_2_	1 mM	215.14 ± 6.92
MnCl_2_	1 mM	145.73 ± 4.01
FeSO_4_	1 mM	144.68 ± 3.10
NiCl_2_	1 mM	65.03 ± 0.98
CuSO_4_	1 mM	6.48 ± 2.04
ZnCl_2_	1 mM	25.13 ± 3.10
BaCl_2_	1 mM	130.19 ± 5.28
FeCl_3_	1 mM	61.03 ± 1.29
EDTA	1 mM	21.20 ± 1.05
EGTA	1 mM	49.55 ± 1.67
DTT	1 mM	71.77 ± 1.03
SDS	3%	2.40 ± 0.23

Moreover, the specific activities of recombinant VxAly7D towards sodium alginate, polyG and polyM were 462.4 ± 0.64, 357.37 ± 0.53, and 441.94 ± 2.46 U/mg, respectively (Fig. 2g), indicating that this enzyme is a bifunctional alginate lyase. Broad substrate adaptability overcomes the limitations of available substrates and facilitates the preparation of monosaccharides.

### End products and action mode of recombinant VxAly7D

**Fig. 3:**
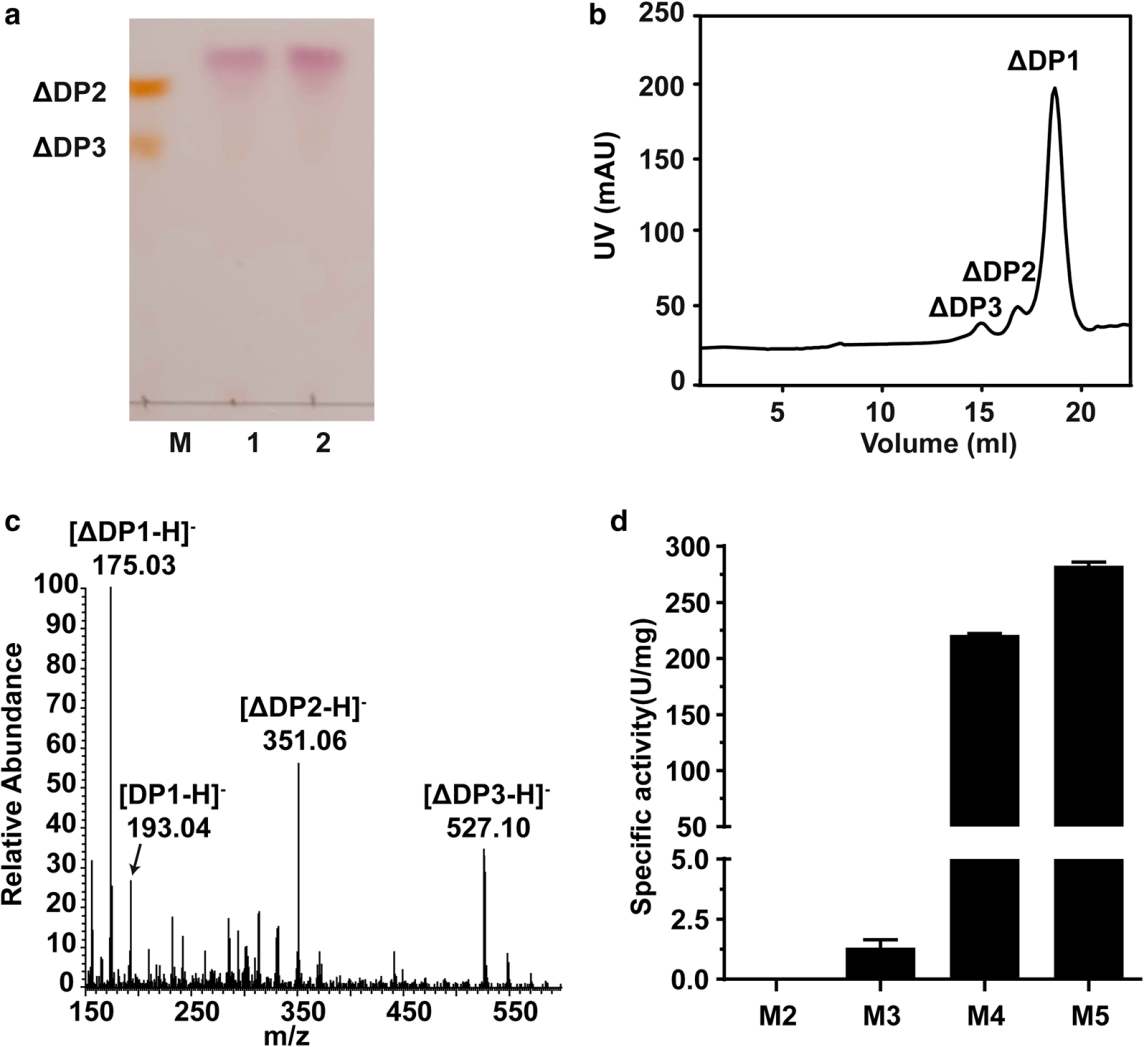
End products and minimal degradation substrate of recombinant VxAly7D. **a** TLC analysis of the end products of recombinant VxAly7D. Lane M, ΔDP2 and ΔDP3; Lane 1 and Lane 2 represent the end products of sodium alginate (3 mg) degraded by 20 U or 100 U recombinant VxAly7D, respectively. **b** The end products of sodium alginate were analysed by gel filtration chromatography. Action of 20 U recombinant VxAly7D on 3 mg sodium alginate at 30 °C for 16 h. The elution volumes were 17.09 ml for monosaccharides, 16.10 ml for disaccharides, and 14.90 ml for trisaccharides. **c** The end products of sodium alginate were analysed by negative ion ESI-MS. **d** Relative activities of recombinant VxAly7D towards M2–M5

The end products of recombinant VxAly7D were prepared by exhaustive degradation with excessive enzyme activity (20 U or 100 U recombinant VxAly7D against 3 mg sodium alginate at 30 °C for 16 h), analysed by TLC and gel filtration chromatography, and identified by negative ion ESI-MS. According to the TLC results, the end products of 20 U and 100 U of recombinant VxAly7D were similar, mainly consisting of monosaccharides with small amounts of di- and trisaccharides (Fig. 3a). The end products of 20 U of recombinant VxAly7D were also analysed by gel filtration chromatography. Sodium alginate was degraded into unsaturated monosaccharides (elution volume of 17.09 ml), unsaturated disaccharides (elution volume of 16.10 ml) and unsaturated trisaccharides (elution volume at 14.90 ml) (Fig. 3b). The contents of unsaturated di- and trisaccharides accounted for approximately 5% of the end products. However, some unsaturated monosaccharides could be converted to DEH, which does not show absorption at 235 nm. Thus, the monosaccharide content constituted not lower than 95% of the total end products. The end products of recombinant VxAly7D were further confirmed by negative ion ESI-MS to be unsaturated monosaccharides (ΔDP1) ([M−H]^−^=175.03), saturated monosaccharides (DP1) ([M−H]^−^=193.04), unsaturated disaccharides (ΔDP2) ([M−H]^−^=351.06) and unsaturated trisaccharides (ΔDP3) ([M−H]^−^=527.09) (Fig. 3c, Additional file 1: Fig. S1a). Moreover, each peak generated in gel filtration chromatography was collected separately, and its molecular weight was identified by negative ion ESI-MS (Additional file 1: Fig. S1b–d).

To determine the minimal degradation substrate of recombinant VxAly7D, recombinant VxAly7D activity towards different oligomannuronates with defined DPs (dimannuronate–pentamannuronate: M2–M5) was measured. Recombinant VxAly7D exhibited no activity towards M2 and presented very weak activity towards M3, with specific activity of 1.32 ± 0.32 U/mg. When the DP of the substrate increased from three to four, the specific activity of recombinant VxAly7D increased to 221 ± 1.36 U/mg (Fig. 3d). The specific activity of recombinant VxAly7D towards M5 was 282.88 ± 2.99 U/mg (Fig. 3d). The above results indicated that the minimal degradation substrate of recombinant VxAly7D was a tetrasaccharide.

**Fig. 4:**
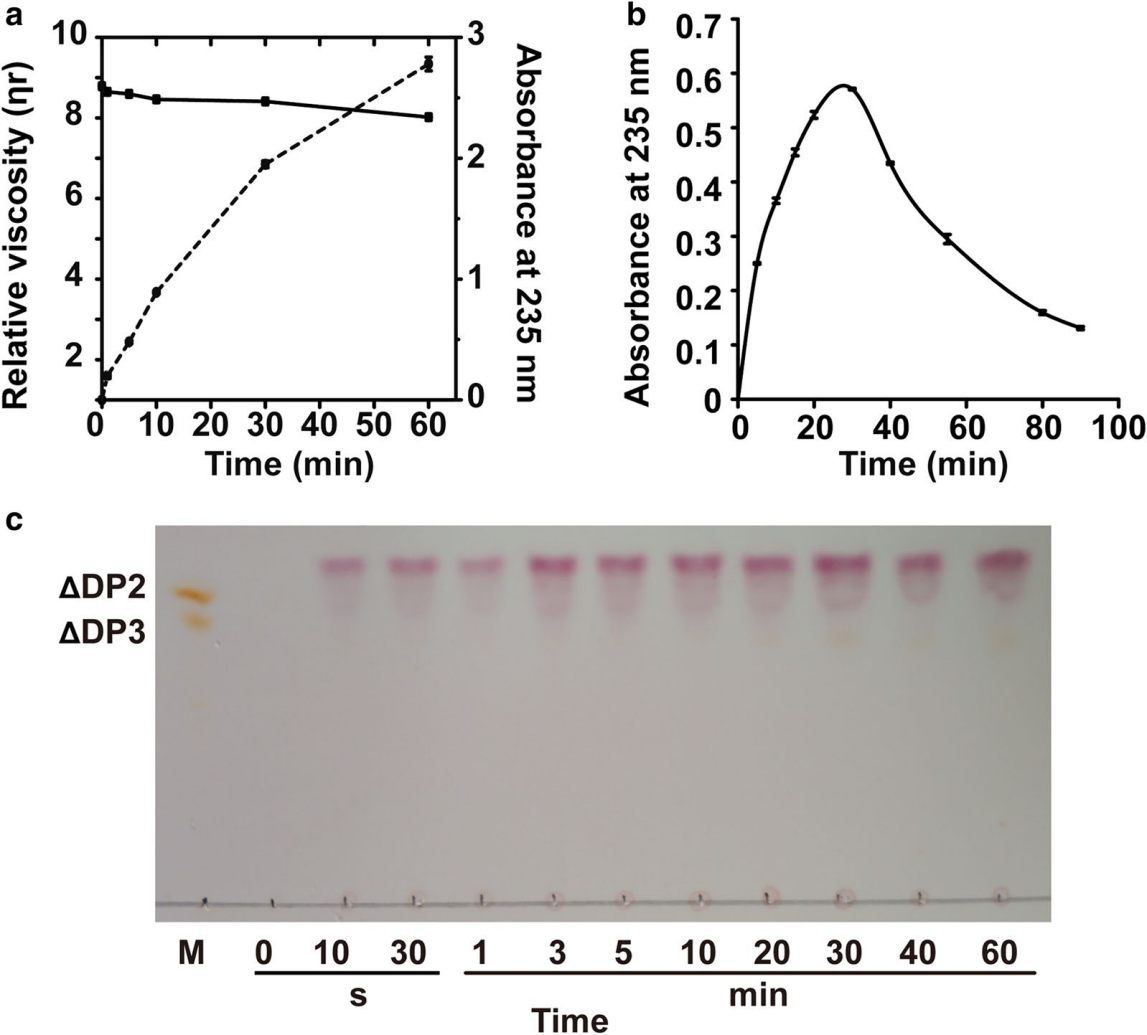
Action mode of recombinant VxAly7D. **a** The action mode was measured according to the changes in absorbance at 235 nm (dotted line) and reduction in viscosity (solid line). One millilitre of enzyme (10 U) was added to 9 ml of substrate solution [0.3% (w/v) high-viscosity sodium alginate, 100 mM NaCl, 20 mM PB, optimal pH], followed by incubation at 30 °C for 0, 1, 5, 10, 30 and 60 min. **b** The time-dependent changes in absorbance at 235 nm of reaction mixtures by recombinant VxAly7D. A total of 12 U enzyme was added to 1 ml substrate solution [0.3% (w/v) sodium alginate, 100 mM NaCl, 20 mM PB, optimal pH], followed by incubation at 30 °C for 0, 5, 15, 20, 30, 40, 55, 80 and 90 min. **c** The time-course of sodium alginate degradation by recombinant VxAly7D was determined by TLC. A total of 5 U enzyme was added to 1 ml substrate solution [0.3% (w/v) sodium alginate, 100 mM NaCl, 20 mM PB, optimal pH], followed by incubation at 30 °C for 0 s, 10 s, 30 s, 1 min, 3 min, 5 min, 10 min, 20 min, 30 min, 40 min, or 60 min

According to the product distribution, recombinant VxAly7D seemed to degrade the substrate via the endo-type action mode to produce oligosaccharides with different DPs; however, the monosaccharide content of the product (≥ 95%) seemed to contradict this speculation. To determine the action mode of recombinant VxAly7D, the viscosity and the absorbance at 235 nm of the reaction mixtures were measured during the enzymatic degradation of high-viscosity sodium alginate. The results showed that A_235_ increased throughout the entire degradation process (Fig. 4a), which indicated that recombinant VxAly7D could continuously degrade sodium alginate to produce unsaturated products. However, the viscosity of the reaction mixture decreased slowly and remained at 85% until 60 min (Fig. 4a), which suggested that recombinant VxAly7D degraded sodium alginate via an exo-type action mode. When the recombinant VxAly7D was incubated with sodium alginate, the absorption curve at 235 nm showed a kinetic in two phases (Fig. 4b). In the first phase, the absorption increased since sodium alginate was degraded by recombinant VxAly7D using β-elimination mechanism. Then the absorption gradually decreased, suggesting the reaction products spontaneously converted to DEH, which did not show absorption at 235 nm. This kinetic change in A_235_ is a typical feature of substrate degradation by exo-type alginate lyase [18]. Moreover, TLC analysis was also used to confirm this conclusion. The time-course analysis of the reaction products showed that monosaccharides and small amounts of di- and trisaccharides were produced from the beginning to the end of the reaction, and no oligosaccharides with other DPs were generated (Fig. 4c). This result also indicated that recombinant VxAly7D is an exo-type alginate lyase.

The above results indicated that recombinant VxAly7D degrades the substrate from the nonreducing end via an exo-type action mode, but cannot degrade oligosaccharides with the DP < 4. Therefore, the end products of recombinant VxAly7D were mostly categorized as ΔDP1 with a small amount of DP1, ΔDP2 and ΔDP3.

### Enzymatic preparation of unsaturated alginate monosaccharide with recombinant VxAly7D

**Fig. 5:**
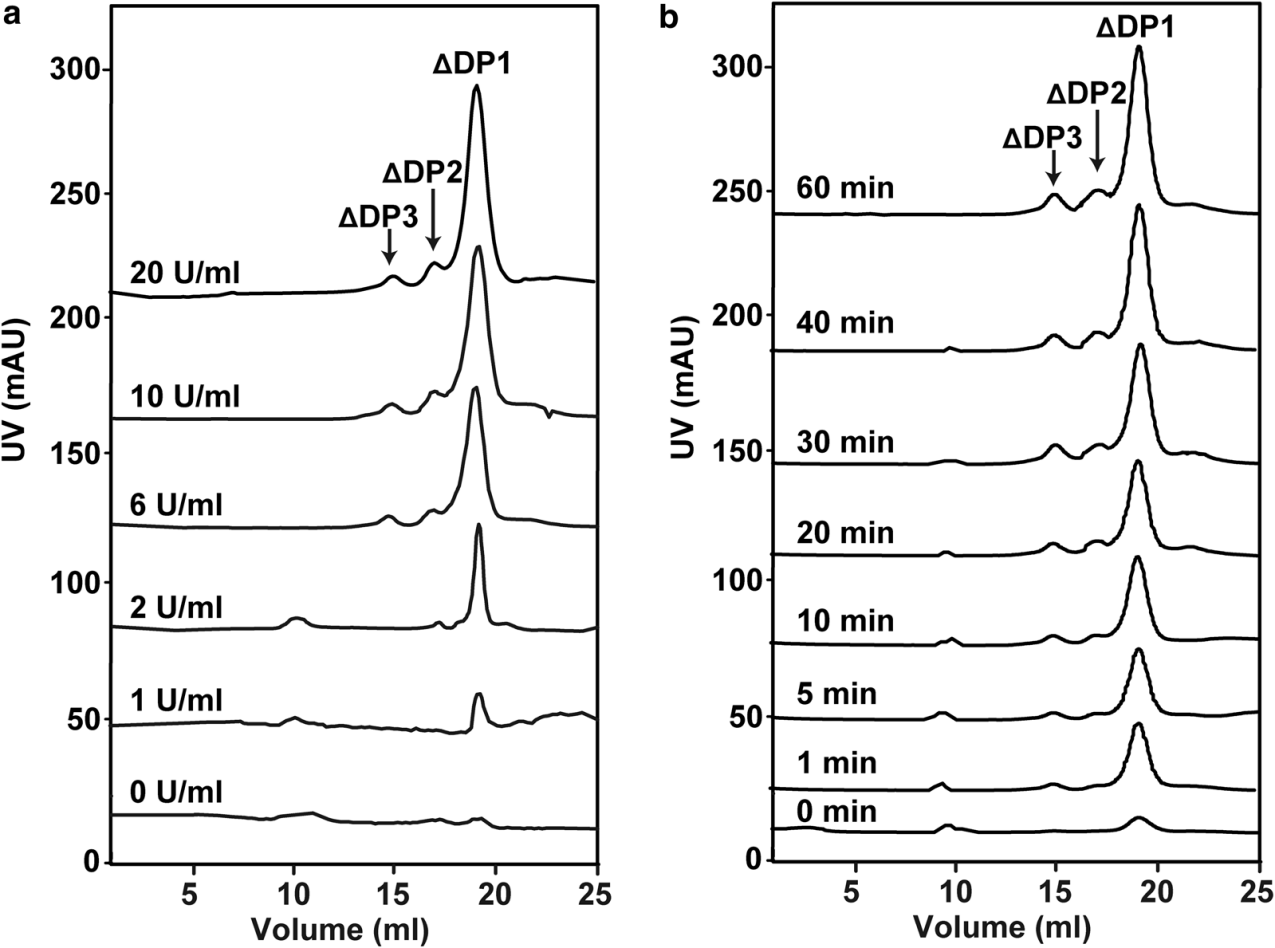
Enzymatic degradation rate of sodium alginate by recombinant VxAly7D. **a** Products of sodium alginate (3 mg/ml) degraded by different amounts (0, 1, 2, 6, 10, 20 U/ml) of recombinant VxAly7D for 1 h. **b** Time-course treatment of sodium alginate (3 mg/ml) using 6 U/ml recombinant VxAly7D at 30 °C for 0, 1, 5, 10, 20, 30, 40 and 60 min

To determine the minimal amount of recombinant VxAly7D capable of rapidly and completely degrading sodium alginate, sodium alginate (3 mg/ml) was digested using recombinant VxAly7D at different concentrations (0, 1, 2, 6, 10, and 20 U/ml) at the corresponding optimal temperature for 1 h. Each enzymatic product (~ 3 mg) was analysed with a Superdex peptide 10/300 GL column and monitored at 235 nm. The results showed that 1 U/ml recombinant VxAly7D could weakly degrade sodium alginate, and 2 U/ml converted 30% of the substrate into unsaturated oligosaccharides (Fig. 5a). Furthermore, the end products of sodium alginate degraded using 6, 10, or 20 U/ml recombinant VxAly7D were similar (Figs. 3b, 5a), indicating that 13 μg (= 6 U) recombinant VxAly7D could convert 3 mg sodium alginate into unsaturated monosaccharides with a yield of 95% in 1 h.

To determine the unsaturated monosaccharide production rate by recombinant VxAly7D, a time-course analysis of sodium alginate degradation was carried out. Sodium alginate (3 mg/ml) was digested using 6 U/ml recombinant VxAly7D for 0–60 min, and the resultant products were collected at 0, 1, 5, 10, 20, 30, 40 and 60 min and analysed by gel filtration chromatography. The amount of the products gradually increased as time increased. The contents of unsaturated monosaccharides in the end products at 1, 5, 10, 20, 30 and 40 min were 37.6%, 47.2%, 59.2%, 76.6% and 85%, respectively (Fig. 5b). Thus, the above results indicated that recombinant VxAly7D could be used to rapidly and highly efficiently degrade sodium alginate and produce unsaturated monosaccharides.

### Homology modelling and structure alignment of VxAly7D

**Fig. 6:**
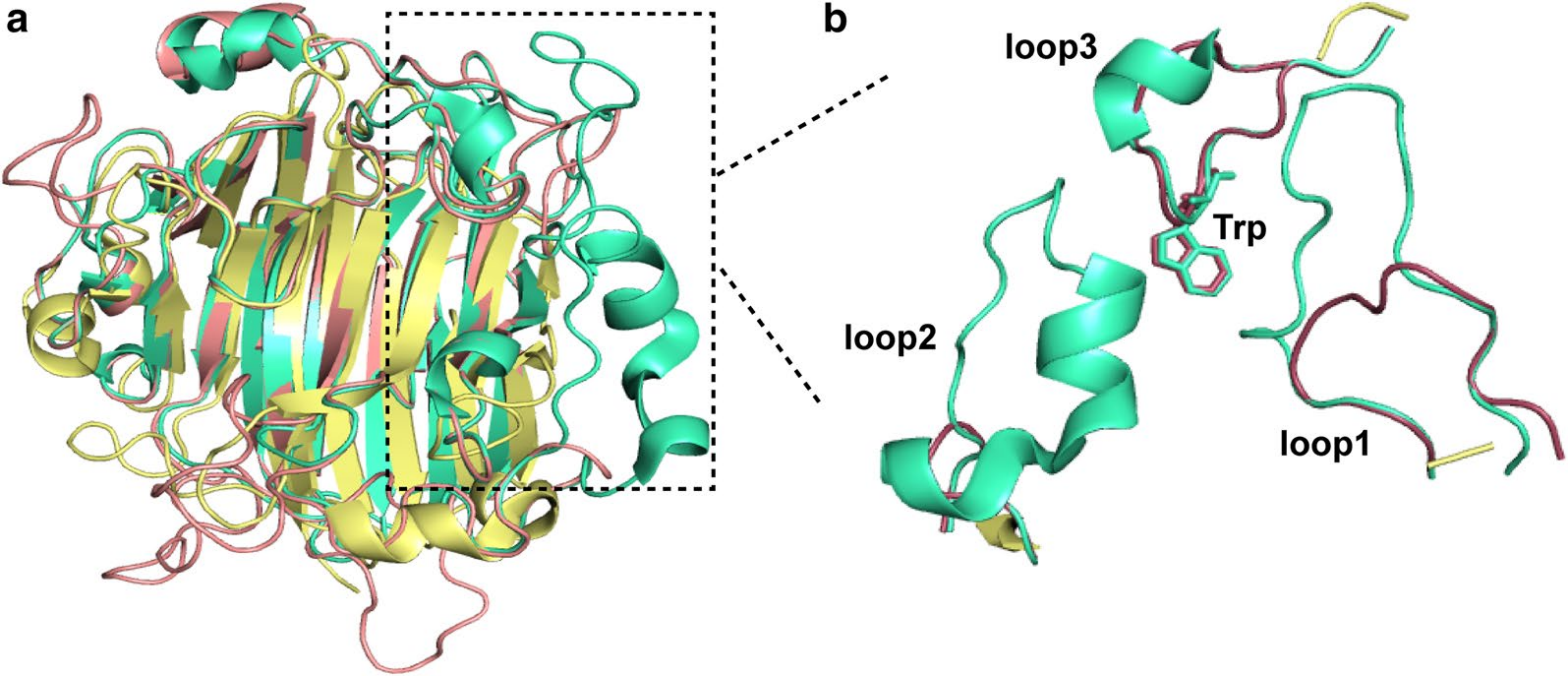
Homology modelling of VxAly7D. **a** The structures of VxAly7D, AlyA5 (PDB ID: 4BE3) and A1-II′ (PDB ID: 2CWS) were performed using PyMOL. VxAly7D, red cartoon; AlyA5, green cartoon; A1-II′, yellow cartoon. **b** Three extra loops of VxAly7D

Among the crystallized PL7 alginate lyases, the VxAly7D showed the highest identity (39.71%) with that of AlyA5 from *Zobellia galactanivorans* Dsij^T^. The structure of VxAly7D was obtained by homologous modelling using AlyA5. VxAly7D has a typical β-jelly roll structure that conforms to the structural features of the PL7 alginate lyases (Fig. 6a). The structure quality of VxAly7D was evaluated by Verify3D [42]. Verify 3D results show that 92.45% of the residues have averaged 3D–1D score of no less than 0.2. Therefore, the structure of VxAly7D constructed by homology modelling is reasonable. The comparison of the structures of the exo-type alginate lyases AlyA5, VxAly7D, and the endo-type alginate lyase A1-II’ from *Sphingomonas* sp. A1 showed that AlyA5 and VxAly7D both exhibit three additional loops (loop1, loop2, and loop3), while A1-II’ does not. However, while loop3 of VxAly7D is consistent with loop3 of AlyA5, loop1 and loop2 of VxAly7D are significantly shorter than loop1 and loop2 of AlyA5. According to previous reports, loop3 might be the critical loop responsible for the action mode of exo-type PL7 alginate lyase [43], in which Trp^295^ of VxAly7D (which corresponds to Trp^313^ of AlyA5) might be a critical amino acid residue because it constitutes a hydrophobic wall obstructing the continuation of the groove [18] (Fig. 6b).

### Construction, purification and biochemical characterization of mutants

According to structural analysis, two mutants, CL3 (loop3-deletion mutant) and W295A (site-directed mutant), were constructed. The purified W295A mutant showed a single band in SDS-PAGE gels with a molecular mass of approximately 36 kDa, as observed for recombinant VxAly7D (Additional file 1: Fig. S2a). The specific activity of purified W295A towards sodium alginate was 74.09 ± 1.53 U/mg, while purified CL3 exhibited almost no activity. The above results indicated that loop3 and Trp^295^ affect the catalysis of the substrate by recombinant VxAly7D.

The biochemical characterization of the W295A mutant was determined. Its optimum pH was 6.6 in PB (Additional file 1: Fig. S2b). The pH stability of the W295A mutant showed that the enzyme was stable at a pH of 6.6–8.0, since approximately 50% of its activity was retained (Additional file 1: Fig. S2c). Compared with other buffers, the W295A mutant retained higher activity and stability in PB buffer. However, compared with recombinant VxAly7D, the W295A mutant exhibited lower pH stability (Fig. 2c, Additional file 1: Fig. S2c). The optimum temperature of the W295A mutant was 30 °C (Additional file 1: Fig. S2d). The W295A mutant exhibited 13%, 33%, and 85% of its highest activity at 0, 10 and 20 °C, respectively. The W295A mutant was stable at 0–30 °C, with approximately 80% of the enzyme activity retained after incubation for 1 h (Additional file 1: Fig. S2e). Similar to recombinant VxAly7D, the W295A mutant still exhibited high activity and stability at low/moderate temperatures (0–30 °C) (Fig. 2d, Additional file 1: Fig. S2d, e). The effects of NaCl (Additional file 1: Fig. S2f), metal ions, surfactants (EDTA and EGTA), detergents (SDS) and reducing agents (DTT) on the activity of the W295A mutant were not significantly different from those of recombinant VxAly7D (Table 1, Additional file 1: Table S1). The substrate preference of the W295A mutant was also consistent with that of recombinant VxAly7D (Fig. 2g, Additional file 1: Fig. S2g).

### Action mode and end products of the W295A mutant

**Fig. 7:**
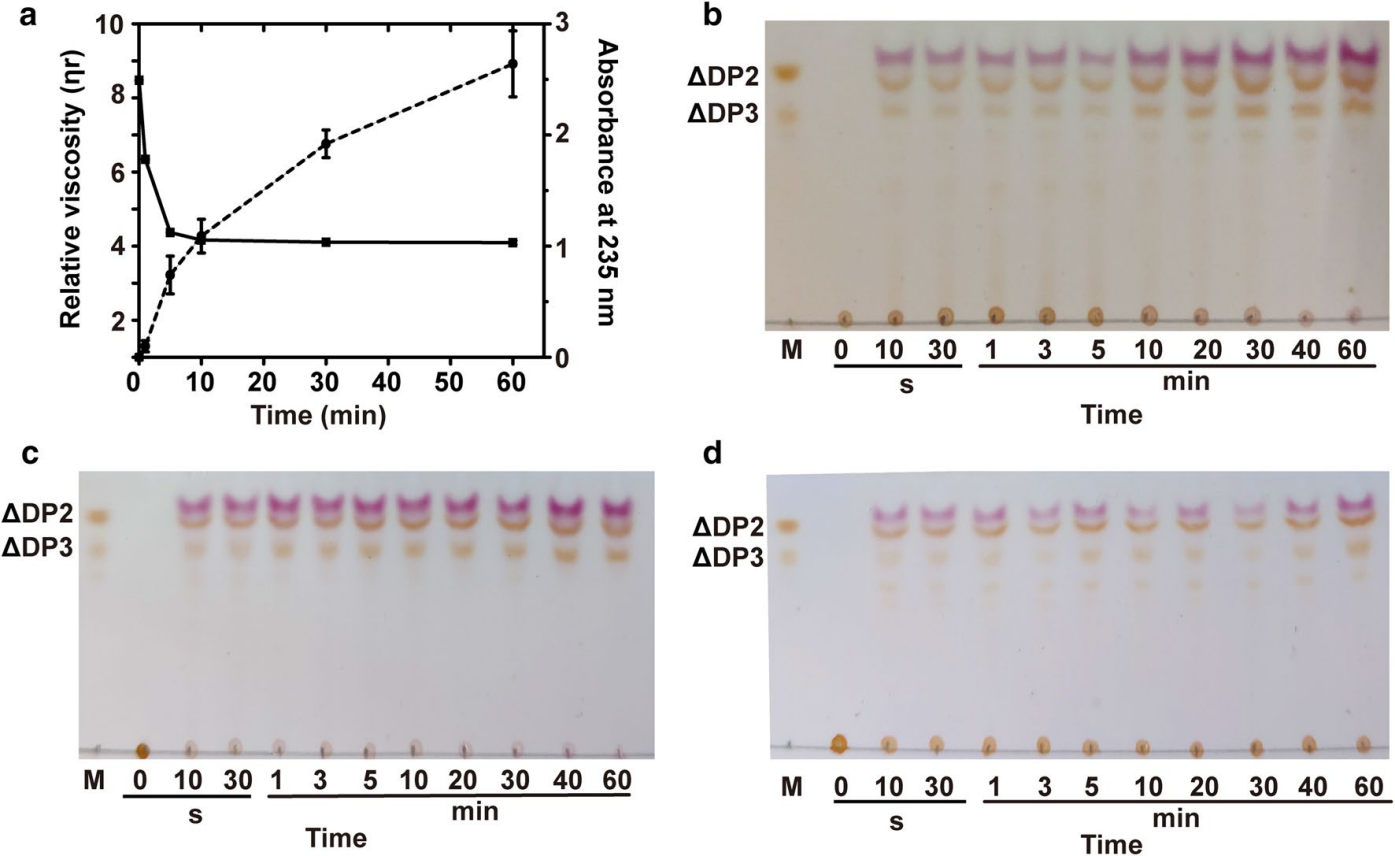
Action mode of the W295A mutant. **a** The action mode was measured according to the changes in absorbance at 235 nm (dotted line) and the reduction in viscosity (solid line). One millilitre of enzyme (10 U) was added to 9 ml of substrate solution [0.3% (w/v) high-viscosity sodium alginate, 100 mM NaCl, 20 mM PB, optimal pH], followed by incubation at 30 °C for 0, 1, 5, 10, 30 and 60 min. **b** The time-course of sodium alginate degradation by the W295A mutant was determined by TLC. A total of 5 U of enzyme were added to 1 ml substrate solution [0.3% (w/v) sodium alginate, 100 mM NaCl, 20 mM PB, optimal pH], followed by incubation at 30 °C for 0 s, 10 s, 30 s, 1 min, 3 min, 5 min, 10 min, 20 min, 30 min, 40 min, or 60 min. **c**, **d** The time-course of polyM and polyG degradation by the W295A mutant were determined by TLC. A total of 3 U of enzyme were added to 1 ml substrate solution [0.3% (w/v) polyM or polyG, 100 mM NaCl, 20 mM PB, optimal pH], followed by incubation at 30 °C for 0 s, 10 s, 30 s, 1 min, 3 min, 5 min, 10 min, 20 min, 30 min, 40 min, or 60 min

We also determined the action mode and the end products of the W295A mutant. The results showed that A_235_ increased throughout the degradation process; however, viscosity decreased rapidly within the first 10 min and decreased slowly in the last 50 min (Fig. 7a), suggesting that the W295A mutant degraded sodium alginate in an endolytic action mode. We also use TLC to further validate the action mode of the W295A mutant. Analysis of the time-course of the reaction products showed that mono-, di-, and trisaccharides, and other oligosaccharides with low DPs were produced at the beginning of the reaction. With the prolongation of reaction time, the amounts of mono-, di- and trisaccharides gradually increased, while those of oligosaccharides with other DPs significantly decreased (Fig. 7b–d). This distribution of the products indicated that the W295A mutant presented both exo- and endo-type action modes. The results showed that Trp^295^ was the key amino acid residue responsible for the exo-type action mode of recombinant VxAly7D.

**Fig. 8:**
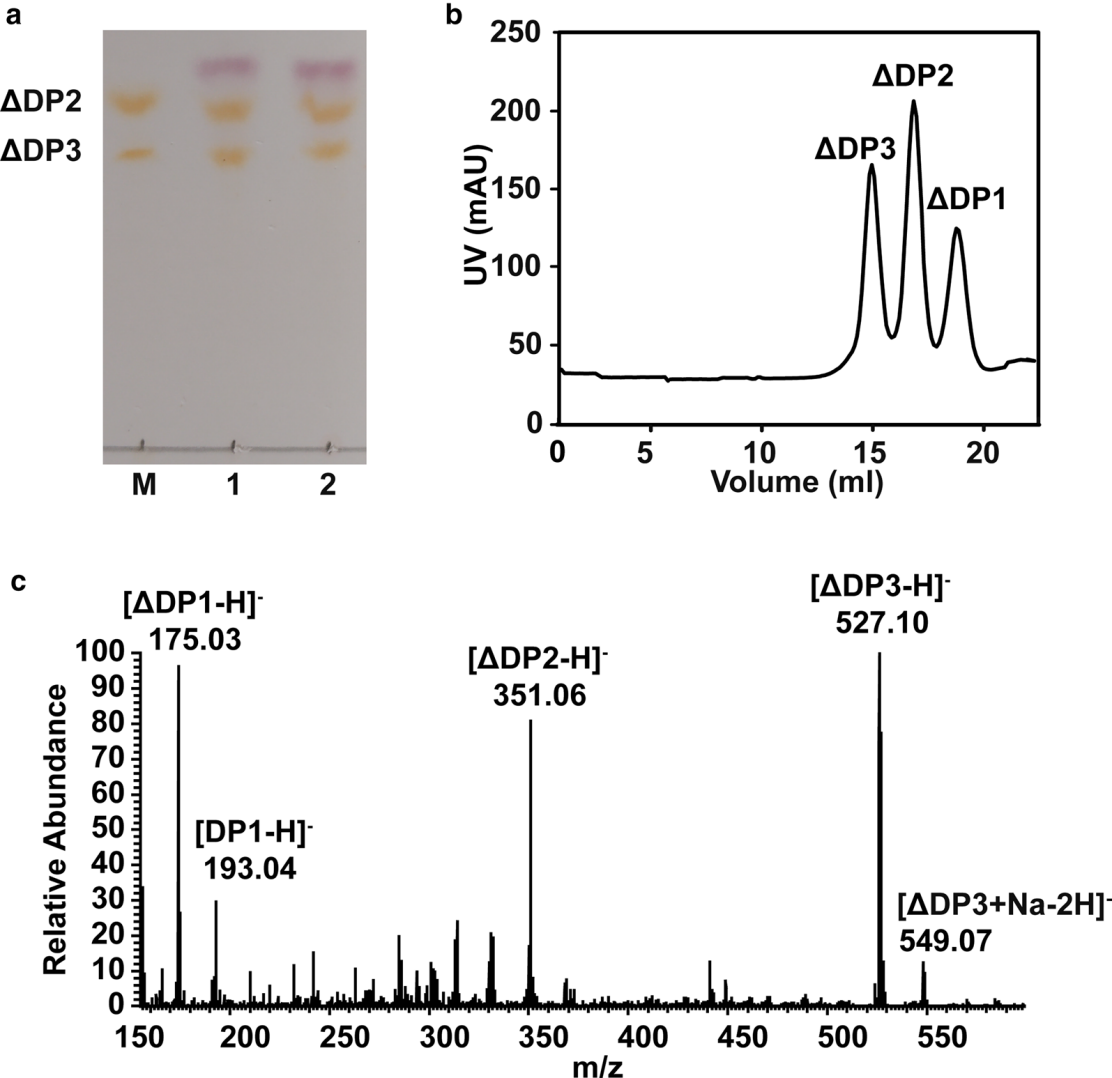
End products of the W295A mutant. **a** TLC analysis of the end products of the W295A mutant. Lane M, ΔDP2 and ΔDP3; lane 1 and lane 2 show the end products of sodium alginate (3 mg) degraded by 20 U or 100 U of the W295A mutant, respectively. **b** The end products of the W295A mutant were analysed by gel filtration chromatography. Action of 20 U of the W295A mutant against 3 mg of sodium alginate at 30 °C for 16 h. **c** The end products of the W295A mutant were analysed by negative ion ESI-MS

According to the TLC analysis, the end products of sodium alginate obtained with 20 U or 100 U of the W295A mutant were similar, and both consisted of mono-, di- and trisaccharides (Fig. 8a). The end products of polyM and polyG were the same with sodium alginate (Additional file 1: Fig. S3). Based on the elution volume and peak areas of gel filtration chromatography, the end products of the W295A mutant were unsaturated mono-, di- and trisaccharides (Fig. 8b). The oligosaccharide products of the W295A mutant were confirmed by negative ion ESI-MS to be ΔDP1 ([M−H]^−^ = 175.03), DP1 (M−H^−^ = 193.04), ΔDP2 ([M−H]^−^ = 351.06) and ΔDP3 ([M−H]^−^ = 527.09) (Fig. 8c, Additional file 1: Fig. S4a). Each peak from the gel filtration chromatography analysis was collected separately, and its molecular weight was identified by negative ion ESI-MS (Additional file 1: Fig. S4b–d).

## Discussion

Nearly half of known alginate lyase sequences have been classified into the PL7 family; however, exo-type PL7 alginate lyases have rarely been reported [18, 39, 44, 45]. In this paper, the exo-type PL7 alginate lyase VxAly7D from *V. xiamenensis* QY104 was cloned and characterized. VxAly7D showed the highest identity (72.54%) to AlyD from *V. splendidus* 12B01. However, compared with AlyD [46], recombinant VxAly7D exhibited lower NaCl dependence and higher metal ion tolerance (Table 2). More importantly, recombinant VxAly7D showed different action mode than AlyD (Fig. 4, Table 2), since AlyD is an endo-type alginate lyase [39, 46]. Both recombinant VxAly7D and AlyA5 from *Zobellia galactanivorans* Dsij^T^ are exo-type alginate lyases [18] belonging to the PL7 family, which cleave the unit at the nonreducing end, but their end products are different. Trisaccharides, which are the minimal degradation substrate of AlyA5, are converted into monosaccharides as the major product and disaccharides as the minor product [18]. However, the minimal degradation substrate of recombinant VxAly7D was found to be tetrasaccharides, which were eventually turned into monosaccharides as the major product and di- and trisaccharides as the minor product (Fig. 3). The above results showed that although VxAly7D shares high identity with AlyD and AlyA5 (Fig. 1a), their biochemical characterization is still significantly different. Furthermore, compared with alginate lyases of the PL7 family with high activity at low/moderate temperatures, recombinant VxAly7D did not exhibit a substrate preference and showed the highest percentage of its maximum activity at 20 °C (Table 3). The above results suggested that VxAly7D has specific characterization as an exo-type alginate lyase of the PL7 family.

**Table 2:** Comparison of biochemical characterization of recombinant VxAly7D and AlyD

Parameter	VxAly7D	AlyD
Origin	*V. xiamenensis* QY104	*V. splendidus* 12B01
Molecular mass	36.46 kDa	36.5 kDa
Optimal pH	7.3	8
Optimal temperature	30 °C	20 °C
Optimal NaCl concentration	100 mM	400 mM
Metal ions		
Inhibition	Cu^2+^, Ni^2+^, Zn^2+^	Cu^2+^, Ni^2+^, Zn^2+^, Fe^2+^, Mg^2+^, Mn^2+^
Promotion	Ca^2+^, Fe^2+^, Mg^2+^, Mn^2+^	Ca^2+^
EDTA	Inhibit	Inhibit
Substrate preference	Sodium alginate (462.40 U/mg)	Alginate (0.5 μM/s/μg)
	polyG (357.37 U/mg)	polyG (0.41 μM/s μg)
	polyM (441.94 U/mg)	polyM (0.25 μM/s μg)
Products	Mono-, di-, trisaccharides	Mono-, di-, pentasaccharides
Action mode	Exo-type	Endo-type
Reference	In this study	[46]

**Table 3:** Comparison of recombinant VxAly7D with other alginate lyases

Enzyme	Source	Family	Specific activity	Optimal temperature	Percent of the maximal activity at 20 °C (%)	Substrate specificity	Products (DP)	Action mode	References
VxAly7D	*Vibrio xiamenensis* QY104	PL7	462.4 U/mg	30	90	Sodium alginate ≈ polyM > polyG	1–3	Exo-type	In this study
AlyA5	*Zobellia galactanivorans* Dsij^T^	PL7	449.3 U/mg	30	No data	PolyG > polyMG > polyM	1–2	Exo-type	[18]
AlyPM	*Pseudoalteromonas* sp. SM0524	PL7	No data	30	52	PolyM > sodium alginate > polyG	2–3	Endo-type	[16]
Algb	*Vibrio* sp. W13	PL7	457 U/mg	30	80	Sodium alginate ≈ polyMG > polyG > polyM	2–5	Endo-type	[62]
AlyA1_PL7_	*Zobellia galactanivorans* Dsij^T^	PL7	No data	30	82	PolyG > polyM	2–4	Endo-type	[18]
TsAly6A	*Thalassomonas* sp. LD5	PL6	189 U/mg	35	73.1	PolyG ≈ alginate > polyM	2–3	Endo-type	[14]
AlyGC	*Glaciecola chathamensis* S18K6^T^	PL6	170 U/mg	30	82.5	PolyG > polyM > sodium alginate	1	Exo-type	[15]
AkAly30	*Aplysia kurodai*	PL14	210 U/mg	55	20	PolyM > alginate > polyMG > polyG	1–3	Endo- and exo-type	[63, 64]
A1-IV	*Sphingomonas* sp. A1	PL15	7.5 U/mg^a^	37	No data	polyM ≈ polyG	1	Exo-type	[65]
AlgL17	*Microbulbifer* sp. ALW1	PL17	No data	35	≤ 65	PolyM > alginate > polyG	1–4	Exo-type	[56]

All of the activity was defined by measuring the increase in absorbance at 235 nm

^a^The activity of A1-IV was calculated according to the method described by Fu et al. [66]

Exo-type alginate lyases cleave the substrate from the ends of the chains [43], resulting in a low degradation rate and monosaccharide production [29–34, 46–49]. The specific activity of recombinant VxAly7D towards sodium alginate was 462.4 ± 0.64 U/mg, which was higher than that of other reported exo-type alginate lyases. Moreover, the unsaturated monosaccharide yield and production rate of recombinant VxAly7D were higher than those of other alginate lyases that function in an exolytic action mode. We found that 13 μg recombinant VxAly7D converted 3 mg sodium alginate to unsaturated monosaccharides with a yield of 95% (Figs. 3b, 5a). Furthermore, gel filtration chromatography analysis showed that sodium alginate could be rapidly degraded to unsaturated monosaccharides with a yield of 37.6% within 1 min (Fig. 5b). The results showed that recombinant VxAly7D was an exo-type alginate lyase that can highly efficiently degrade sodium alginate to produce unsaturated monosaccharides. In addition, due to the low activity of reported exo-type alginate lyases, a method for applying the synergistic action of endo-type and exo-type alginate lyases was established to obtain a high yield of monosaccharides [4, 6, 38]. Alg7A/Alg7K from *Saccharophagus degradans* co-displaying yeast has been reported to exhibit high sodium alginate-degrading activity, producing 1.98 g/l of reducing sugars in 1 h [4]. However, its polysaccharide conversion rate was only 19.8%, which was far lower than that of recombinant VxAly7D. When 0.27 μg of the endo-type alginate lyase AlyPB1 and 4.5 μg of AlyPB2 from *Photobacterium* sp. FC615 acted on alginate together, and the yield of monosaccharides was dramatically increased approximately sevenfold compared with that resulting from the action of AlyPB2 applied alone for 10 min [38]; however, it was still lower than that of recombinant VxAly7D at 37.6% at 1 min. Therefore, whether recombinant VxAly7D is used alone or in combination with endo-type alginate lyases, it increases the monosaccharides yield and productivity.

In addition to enzyme activity, substrate specificity plays a key role in monosaccharide production by exo-type alginate lyases. Many exo-type alginate lyases present a significant substrate preference. For example, AlyGC from *Glaciecola chathamensis* S18K6^T^ [15], OalS6 from *Shewanella* sp. Kz7 [50], AlyA5 from *Zobellia galactanivorans* Dsij^T^ [18], CaAly4 and CaAly5 from *Cellulophaga algicola* DSM 14237 [44], and OalC6 from *Cellulophaga* sp. SY116 prefer polyG blocks [30], while HdAlex from *Haliotis discus hannai* [34], OalC17 from *Cellulophaga* sp. SY116 [30] prefer polyM blocks. Although the combination of exo-type alginate lyases with different preferences could compensate for the disadvantage [30], multi-enzyme degradation is somewhat more complicated than single-enzyme degradation. Exo-type alginate lyases with substrate preference are limited by the substrate blocks, which affects the industrial production of monosaccharide. As a bifunctional alginate lyase, recombinant VxAly7D exhibited similar activity towards polyM and polyG that was close to the activity towards sodium alginate, indicating that it could effectively degrade substrates of any block and drive the production process (Fig. 2g).

Mesophilic alginate lyases present optimal temperatures (usually 40–50 °C) and are generally stable below 40 °C [51–54]. Compared with mesophilic homologs, alginate lyases, which exhibit higher activity and stability at low/moderate temperatures, provide economic benefits by reducing the energy cost of the production process [55]. These enzymes usually show high activity below 35 °C and are stable below 30 °C [27, 56–58]. The optimal temperature of recombinant VxAly7D was 30 °C. Even at 0 °C, recombinant VxAly7D exhibited 21% of its highest activity (Fig. 2d). Compared with exo-type alginate lyases from different PL families, recombinant VxAly7D still presented high activity (415.8 U/mg) in degrading substrates with different conformations to obtain monosaccharides at 20 °C (Table 3). Therefore, recombinant VxAly7D is more suitable for reaction conditions performed at room temperature without additional energy consumption.

According to the CAZy database, AlyA5 from *Zobellia galactanivorans* Dsij^T^ is the only exo-type alginate lyase with available structural information and characterization results among PL7 family. The structural features leading to the exolytic action mode were predicted to be the three large additional loops (Trp^197^-Asp^217^, Ser^257^-Glu^284,^ and Gly^304^-Asp^318^) closed up one end of the catalytic groove [18, 43]. In particular, Trp^313^ is located close to three conserved catalytic residues, forming a hydrophobic wall that obstructed the continuation of the catalytic groove [18]. The above three large additional loops of AlyA5 corresponded to VxAly7D as loop1, loop2, and loop3 (Fig. 6b). The results showed that loop1 and loop2 of VxAly7D were obviously shorter than those of AlyA5 (Fig. 6b). However, recombinant VxAly7D still exhibited exolytic activity (Fig. 4), indicating that loop3 might be the key structural feature leading to the exo-type action mode. The results obtained for the loop3-deletion mutant CL3 demonstrated that loop3 might not only affect the exo-type action mode of VxAly7D but, more importantly, might also affect its catalytic ability. Since the viscosity change associated with an endo-type action mode is more obvious than that for the exo-type action mode, the viscosity change cannot determine the action mode when both endo- and exo-type action modes occur [59]. Furthermore, the TLC analysis result of the time-course suggested that the W295A mutant, while retaining the exo-type action mode, also exhibited an endo-type action mode, resulting in the occurrence of oligosaccharides with other DPs during the degradation process (Fig. 7b–d). The change in the action mode of the W295A mutant indicated that the hydrophobic power generated by Trp was the main factor affecting the exo-type action mode of PL7 alginate lyases.

## Conclusions

In this study, we cloned and characterized an exo-type PL7 alginate lyase, VxAly7D, from the marine bacterium *V. xiamenensis* QY104. Recombinant VxAly7D is a bifunctional exo-type alginate lyase that exhibits high specific activity towards sodium alginate, polyG and polyM. For further industrial applications, recombinant VxAly7D exhibited high activity and stability at low/moderate temperatures. Importantly, recombinant VxAly7D could quickly and highly efficiently convert sodium alginate to unsaturated monosaccharides. A total of 13 μg recombinant VxAly7D could convert 3 mg sodium alginate to unsaturated monosaccharides in 1 min with a yield of 37.6%, and the yield reached 95% in 1 h. Therefore, due to its high activity at low/moderate temperatures and broad substrate specificity, VxAly7D may become a potential tool enzyme for the production of unsaturated monosaccharides with low energy consumption, providing precursors for obtaining DEH. In addition, we demonstrated the mechanism of exolytic action mode of VxAly7D, promoting our understanding of the mechanism of alginate polysaccharide metabolism in marine ecosystems.

## Methods

### Materials and strains

Sodium alginate was obtained from the Bright Moon Seaweed Group (originating from brown algae, 600 cPs; Qingdao, China). High-viscosity sodium alginate was purchased from Sigma-Aldrich (originating from brown algae, 3330 cPs; St. Louis, MO, USA). PolyM and polyG (6–8 kDa) were purchased from Qingdao BZ Oligo Biotech Co., Ltd., Qingdao, China. The chemical reagents were all of analytical grade. Restriction enzymes and the pET-24a (+) vector plasmid were obtained from Takara Co., Ltd., Dalian, China. The high-fidelity DNA polymerase used for PCR was procured from Vazyme Biotech Co. Ltd., Nanjing, China. Primer synthesis and DNA sequencing were performed at the Beijing Genomics Institute (BGI).

*Escherichia coli* DH5α was used for DNA cloning. *E. coli* BL21 (DE3) was used for recombinant protein expression. The marine bacterium *V. xiamenensis* QY104, isolated from seawater from Qingdao coast, was cultured at 25 °C in medium (pH 7.0) containing (w/v) 2.5% NaCl, 0.25% casamino acids, 0.1% KCl, 0.5% MgSO_4_·7H_2_O, 0.02% CaCl_2_, 0.15% NaH_2_PO_4_, 0.2% NaNO_3_, 0.002% FeSO_4_·7H_2_O and 0.3% sodium alginate (1.5% agar in solid medium). The bacterial genome was sequenced and assembled by Novogene (Beijing, China).

### Sequence analysis and homology modelling of VxAly7D

The signal peptide was predicted using the SignalP 5.0 server (http://www.cbs.dtu.dk/services/SignalP/). The theoretical molecular weight (Mw) and isoelectric point (pI) were calculated using the Compute pI/Mw tool (https://web.expasy.org/compute_pi/). The amino acid sequence alignment between VxAly7D and crystallized PL7 alginate lyases was obtained using ClustalW and further aligned with ESPript 3.0 (http://espript.ibcp.fr/ESPript/cgi-bin/ESPript.cgi). The phylogenetic tree was constructed using MEGA 7.0 via the neighbour-joining method. The three-dimensional structure of VxAly7D was modelled with SWISS-MODEL (https://swissmodel.expasy.org/) using AlyA5 (PDB ID: 4BE3) from *Zobellia galactanivorans* Dsij^T^ as the template and was superimposed and described using PyMOL. The quality of the structure of VxAly7D was verified by Verify3D (https://servicesn.mbi.ucla.edu/Verify3d/). The sequence of VxAly7D was submitted to GenBank under Accession number MN704375.

### Cloning, overexpression and purification of recombinant VxAly7D and VxAly7D mutants

The *vxaly7D* gene without the sequence encoding the signal peptide was amplified from *V. xiamenensis* QY104 genomic DNA by PCR. Using the restriction enzyme sites *Nde* I and *Xho* I, the PCR product was ligated into the expression vector pET-24a (+), obtaining a recombinant VxAly7D protein with a C-terminal (His)_6_-tag. The W295A and CL3 mutants were generated via overlap extension PCR with the pET-24a (+) harbouring the VxAly7D sequence as the template. The primers used for these purposes are shown in Additional file 1: Table S2.

The verified recombinant plasmids were transformed into the *E. coli* BL21 (DE3) expression strain and cultured overnight at 37 °C on LB plates supplemented with 80 μg/ml kanamycin. Single colonies were inoculated into fresh kanamycin-containing LB medium and cultured for 16 h at 37 °C at 160 rpm, and then transferred to 100 ml kanamycin-containing LB medium and cultured at 160 rpm at 37 °C until the OD_600_ reached 0.5. Isopropyl-*β*-d-thiogalactopyranoside (IPTG) was added to a final concentration of 0.1 mM, followed by incubation for an additional 39 h at 18 °C.

Cell pellets were harvested at 12,000 rpm at 4 °C for 15 min, then resuspended in binding buffer [20 mM Na_2_HPO_4_–NaH_2_PO_4_ buffer (PB), 500 mM NaCl, pH 7.3] and crushed with a high-pressure crusher (JNBIO, Guangzhou China). Cell debris was removed by centrifugation at 12,000 rpm for 20 min, and the supernatant was collected as the crude enzyme solution. The crude enzyme solution was purified by ÄKTA Fast Protein Liquid Chromatography (FPLC) with a 5-ml Ni–NTA Sepharose column (GE Healthcare, Stamford, USA) at a flow rate of 2 ml/min. Binding buffer was used to remove unbound protein. The proteins were eluted in fractions containing 25, 100, 250 and 500 mM imidazole in washing buffer, and the target protein was successfully recovered at the imidazole concentration of 250 mM. The purity and molecular mass of the protein were analysed by 12.5% (w/v) sodium dodecyl sulfate-polyacrylamide gel electrophoresis (SDS-PAGE).

### Enzymatic activity assay

The reaction mixture included 0.1 ml of purified enzyme solution and 0.9 ml of 0.3% sodium alginate (w/v) in PB containing 100 mM NaCl (pH 7.3) and was incubated for 10 min at 30 °C. The alginate lyase activities of recombinant VxAly7D and the mutants were assayed by measuring the increase in the absorbance at 235 nm (A_235_) for the unsaturated product. One unit (U) of activity was defined as an increase in the A_235_ value of 0.1 per min. The protein concentration was measured with BCA Protein Quantification Kits (Vazyme Biotech Co. Ltd., Nanjing, China).

### Biochemical characterization of recombinant VxAly7D and the W295A mutant

Buffers with different pH values were used to measure the optimal pH and pH stability, including 50 mM Na_2_HPO_4_–citric acid buffer (pH 3.0–8.0), 50 mM Na_2_HPO_4_–NaH_2_PO_4_ buffer (pH 6.0–7.6), 50 mM Tris–HCl buffer (pH 7.05–8.95) and 50 mM Gly–NaOH buffer (pH 8.6–10.6). The optimal pH of recombinant VxAly7D and the W295A mutant was determined in each buffer at 30 °C and assayed as described above. For the determination of pH stability, residual activity was detected after the incubation of recombinant VxAly7D and the W295A mutant in different buffers for 12 h at 4 °C. The optimal temperature was measured by incubation for 10 min at the optimal pH at various temperatures (0, 10, 20, 30, 40, 50 and 60 °C). Temperature stability was determined by measuring residual activity after incubation for 1 h at different temperatures. In addition, the temperature stability was also investigated by measuring the residual activity after incubating the recombinant VxAly7D at 20 °C and 30 °C for 0–24 h. Enzyme activity was measured at the optimal pH in 0.3% (w/v) sodium alginate containing 0, 0.1, 0.25, 0.5, 0.75 or 1.0 mM NaCl to determine NaCl dependence. For the investigation of substrate specificity, 0.3% (w/v) sodium alginate, polyM and polyG were used as substrates.

### Action mode and end products of recombinant VxAly7D and the W295A mutant

To determine the action mode of recombinant VxAly7D and the W295A mutant, 1 ml enzyme (10 U) was added to 9 ml substrate solution [0.3% (w/v) high-viscosity sodium alginate, 100 mM NaCl, 20 mM PB, optimal pH], followed by incubation at 30 °C for 0, 1, 5, 10, 30 and 60 min. The changes in viscosity and A_235_ were measured with a viscometer and UV detector. In addition, to detect the absorption change of the products of recombinant VxAly7D at 235 nm, 12 U recombinant VxAly7D was added to 1 ml substrate solution [0.3% (w/v) sodium alginate, 100 mM NaCl, 20 mM PB, optimal pH], followed by incubation at 30 °C for 0, 5, 15, 20, 30, 40, 55, 80 and 90 min. The changes in A235 were measured with a UV detector.

Then, the degradation products obtained from the time-course analysis of recombinant VxAly7D and the W295A mutant were further characterized using thin-layer chromatography (TLC) as previously reported [60]. A total of 5 U enzyme was added to 1 ml substrate solution [0.3% (w/v) substrate in 20 mM PB supplemented with 100 mM NaCl, optimal pH], followed by incubation at 30 °C for 0 s, 10 s, 30 s, 1 min, 3 min, 5 min, 10 min, 20 min, 30 min, 40 min, and 60 min. In brief, the reaction was stopped by boiling for 5 min, and the degradation products were separated in TLC aluminium silica gel plates (Merck, Germany) developed with *n*-butanol/formic acid/water (4:6:1, v:v:v). They were visualized via the 1,3-naphthalenediol staining method as previously reported [60, 61].

To obtain the end products, each enzyme was added to 1 ml of substrate solution [0.3% (w/v) sodium alginate in 20 mM PB supplemented with 100 mM NaCl, optimal pH] to a final concentration of 20 U/ml or 100 U/ml, followed by culture at 30 °C for 16 h. To determine the components of the end products, TLC analysis of the end products was carried out according to the above method. Then, FPLC with a Superdex peptide 10/300 gel filtration column (GE Health, USA) was applied. The flow rate of the mobile phase (0.2 M NH_4_HCO_3_) was 0.2 ml/min, and the absorbance was monitored at 235 nm. Additionally, the end products mixed with acetonitrile 1:1 (v/v) were detected from 150 to 2000 m/z by negative ion electrospray ionization mass spectrometry (ESI-MS) to determine their molecular weight.

## Supplementary information

**Additional file 1: Table S1.** Effect of metal ions, chelators, and detergents on the W295A mutant activity. **Table S2.** PCR primers for the recombinant VxAly7D and the W295A and CL3 mutants. **Fig. S1.** Negative ion ESI-MS analysis of the end products of recombinant VxAly7D. **Fig. S2.** Purity analysis and biochemical characterization of the W295A mutant. **Fig. S3.** TLC analysis of the end products of the W295A mutant towards polyM and polyG. **Fig. S4.** Negative ion ESI-MS analysis of the end products of the W295A mutant.

## Data Availability

Not applicable.

## Abbreviations

Δ: 4-Deoxy-l-*erythro*-5-hexoseulose uronic acid
DP: Degree of polymerization
G: Guluronate
M: Mannuronate
PolyG: Polyguluronate
PolyM: Polymannuronate
SDS-PAGE: Sodium dodecyl sulfate-polyacrylamide gel electrophoresis
PL family: Polysaccharide lyase family
PB: Na_2_HPO_4_–NaH_2_PO_4_ buffer
ΔDP1: Unsaturated monosaccharides
ΔDP2: Unsaturated disaccharides
ΔDP3: Unsaturated trisaccharides
DP1: Saturated monosaccharides
CAZy database: Carbohydrate-Active enZYmes database
DEH: 4-Deoxy-L-*erythro*-5-hexoseulose uronate
KDG: 2-Keto-3-deoxygluconate
ED pathway: Entner–Doudoroff pathway
EDTA: Ethylenediaminetetraacetic acid
DTT: Dithiothreitol
EGTA: Ethylenebis (oxyethylenenitrilo) tetraacetic acid
FPLC: Fast protein liquid chromatography
IPTG: Isopropyl-*β*-d-thiogalactopyranoside
