# Supplementary material for: Biochemical characteristics and molecular mechanism of an exo-type alginate lyase VxAly7D and its use for the preparation of unsaturated monosaccharides
Source: Biotechnol Biofuels. 2020 Jun 1;13:99. doi: 10.1186/s13068-020-01738-4 (PMC7268478; doi:10.1186/s13068-020-01738-4)
Supplement: Supplementary file 1 — Additional file 1: Table S1. Effect of metal ions, chelators, and detergents on the W295A mutant activity. Table S2. PCR primers for the recombinant VxAly7D and the W295A and CL3 mutants. Fig. S1. Negative ion ESI-MS analysis of the end products of recombinant VxAly7D. Fig. S2. Purity analysis and biochemical characterization of the W295A mutant. Fig. S3. TLC analysis of the end products of the W295A mutant towards polyM and polyG. Fig. S4. Negative ion ESI-MS analysis of the end products of the W295A mutant. [file 13068_2020_1738_MOESM1_ESM.docx]

**Additional File**

**Table S1 Effect of metal ions, chelators, and detergents on the W295A mutant activity.**

| Reagents added | Concentration | Relative activity (%) |
| --- | --- | --- |
| None | 0 mM | 100.00±2.03 |
| LiCl | 1 mM | 158.17±8.59 |
| NH_4_Cl | 1 mM | 110.13±4.17 |
| MgCl_2_ | 1 mM | 106.62±6.82 |
| CaCl_2_ | 1 mM | 204.94±5.57 |
| MnCl_2_ | 1 mM | 133.61±1.32 |
| FeSO_4_ | 1 mM | 127.61±4.89 |
| NiCl_2_ | 1 mM | 20.43±1.51 |
| CuSO_4_ | 1 mM | 10.35±2.21 |
| ZnCl_2_ | 1 mM | 2.41±0.87 |
| BaCl_2_ | 1 mM | 96.97±5.60 |
| FeCl_3_ | 1 mM | 43.41±4.36 |
| EDTA | 1 mM | 35.52±0.01 |
| EGTA | 1 mM | 76.63±1.32 |
| DTT | 1 mM | 93.95±0.37 |
| SDS | 3% | 7.94±0.33 |

**Table S2 PCR primers for the recombinant VxAly7D and the W295A and CL3 mutants.**

| Enzyme | Primer sequences (5’-3’) |
| --- | --- |
| VxAly7D | Upstream: taagaaggagatatacatatgAGCGCTCTAGACAACGGTATTTC |
|  | Downstream: gtggtggtggtggtgctcgagTTTCCCATTGAGTTTTAAGGCAG |
| W295A | Upstream: GAAGCTCATCCAGTCGCAGGACCTGGGTGCGC |
|  | Downstream: GCGCACCCAGGTCCTGCGACTGGATGAGCTTC |
| CL3 | Upstream 1: taagaaggagatatacatatgAGCGCTCTAGACAACGGTATTTC |
|  | Downstream 1: AAAGTCACCGGTACCACTACATTGACCATA |
|  | Upstream 2: TATGGTCAATGTAGTGGTACCGGTGACTTT |
|  | Downstream 2: gtggtggtggtggtgctcgagTTTCCCATTGAGTTTTAAGGCAG |


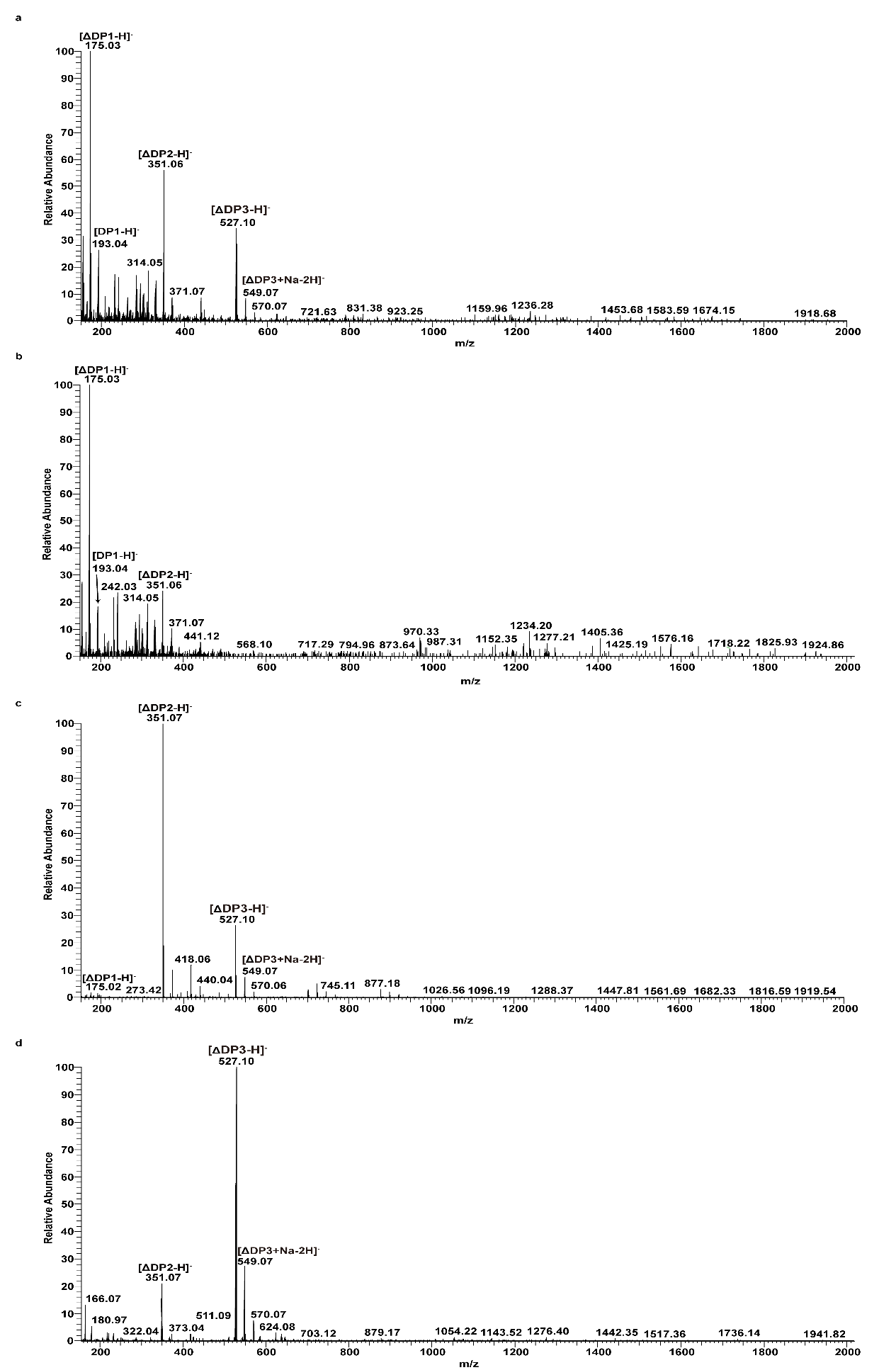


**Fig. S1 Negative ion ESI-MS analysis of the end products of recombinant VxAly7D**. a, total end products; b, monosaccharides; c, disaccharides; d, trisaccharides.


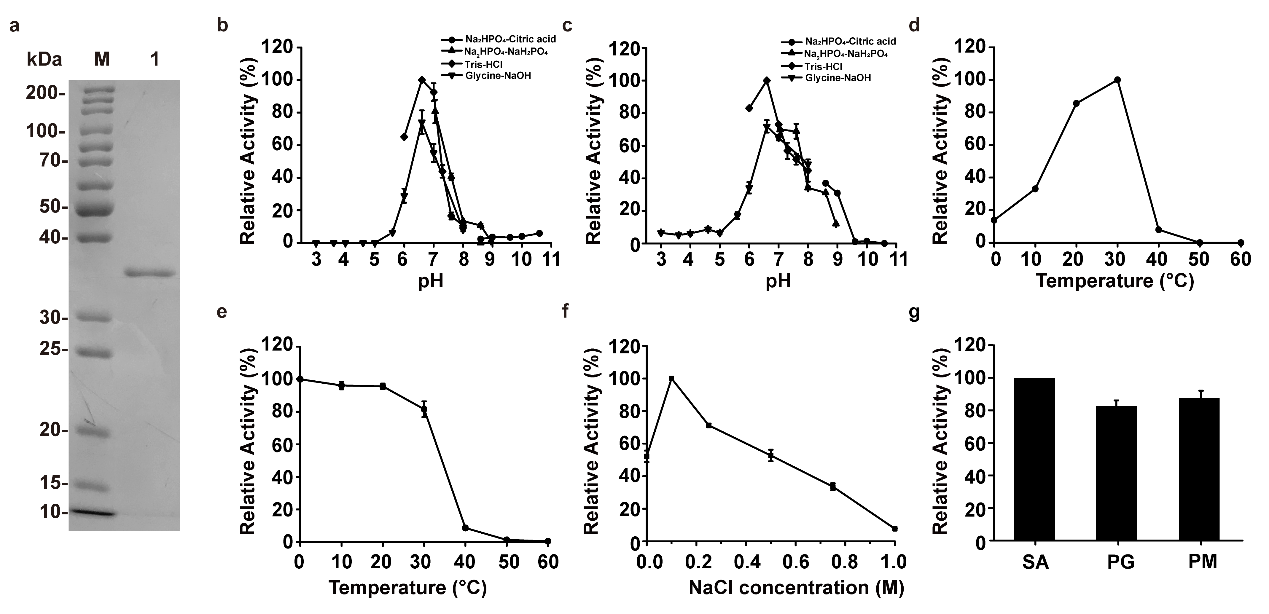


**Fig. S2 Purity analysis and biochemical characterization of the W295A mutant.** a, Purified W295A was resolved by 12.5% (w/v) SDS-PAGE. Lane M, Pageruler unstained protein ladder (Thermo Scientific, USA); Lane 1, purified W295A. b, The optimum pH was determined by measuring the activity at 30 °C in 50 mM buffer (Na_2_HPO_4_‐citric acid, Na_2_HPO_4_‐NaH_2_PO_4_, Tris‐HCl, and Gly‐NaOH) with different pH values. The maximum specific activity was 74.09±1.53 U/mg. c, pH stability. The residual activity was measured at 30 °C in PB (pH 7.3) after incubation at 4 °C for 12 h. The initial specific activity was 71.54±0.63 U/mg. d, The optimal temperature was determined by measuring the activity at 0-60℃. The maximum specific activity was 74.09±1.53 U/mg. e, Temperature stability was determined by measuring the residual activity at 30 °C after incubation at 0-60 °C for 1 h. The initial specific activity was 69.55±2.34 U/mg. f, Effect of the NaCl concentration on the enzymatic activity of the W295A mutant. The maximum specific activity was 74.09±1.53 U/mg. g, Substrate specificity towards sodium alginate (SA), polyM (PM) and polyG (PG). The maximum specific activity was 74.09±1.53 U/mg.


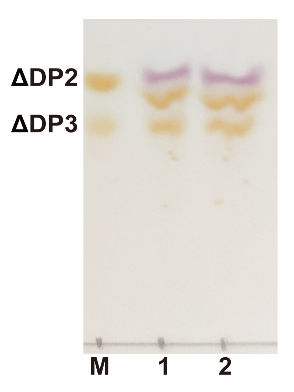


**Fig. S3 TLC analysis of the end products of the** **W295A mutant towards polyM and polyG**. Lane M, ΔDP2 and ΔDP3; Lane 1 and Lane 2 represent the end products of polyG and polyM (3 mg) degraded by 20 U W295A mutant, respectively.


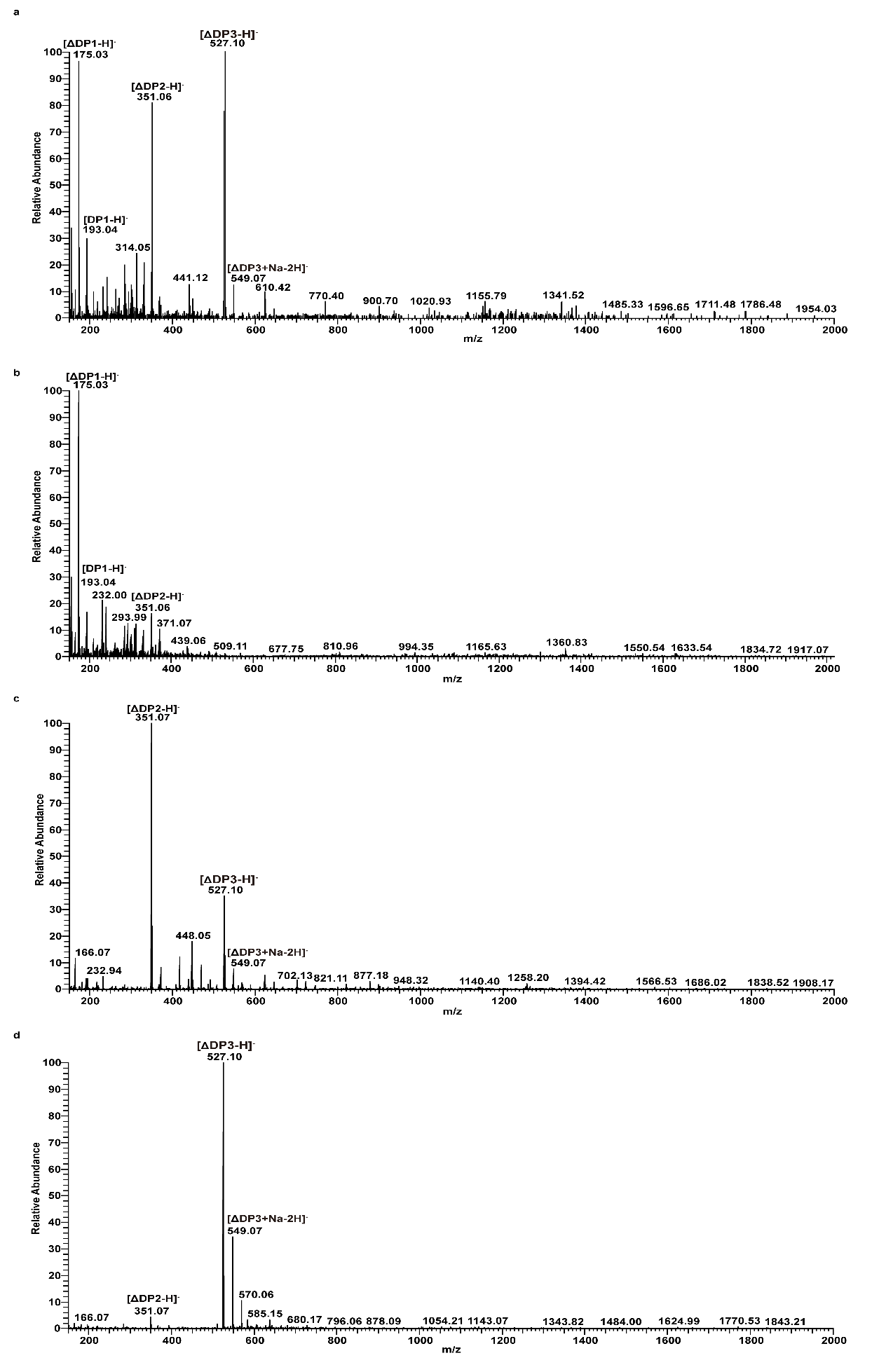


**Fig. S4 Negative ion ESI-MS analysis of the end products of the W295A mutant.** a, total end products; b, monosaccharides; c, disaccharides; d, trisaccharides.
